# First Direct Evidence of Chalcolithic Footwear from the Near Eastern Highlands

**DOI:** 10.1371/journal.pone.0010984

**Published:** 2010-06-09

**Authors:** Ron Pinhasi, Boris Gasparian, Gregory Areshian, Diana Zardaryan, Alexia Smith, Guy Bar-Oz, Thomas Higham

**Affiliations:** 1 Department of Archaeology, University College Cork, Cork, Ireland; 2 Institute of Archaeology and Ethnology, National Academy of Sciences, Yerevan, Armenia; 3 Cotsen Institute of Archaeology, University of California Los Angeles, Los Angeles, California, United States of America; 4 Department of Anthropology, University of Connecticut, Storrs, Connecticut, United States of America; 5 Zinman Institute of Archaeology, University of Haifa, Haifa, Israel; 6 Oxford Radiocarbon Accelerator Unit, University of Oxford, Oxford, United Kingdom; University of Oxford, United Kingdom

## Abstract

In 2008, a well preserved and complete shoe was recovered at the base of a Chalcolithic pit in the cave of Areni-1, Armenia. Here, we discuss the chronology of this find, its archaeological context and its relevance to the study of the evolution of footwear. Two leather samples and one grass sample from the shoe were dated at the Oxford Radiocarbon Accelerator Unit (ORAU). A third leather sample was dated at the University of California-Irvine Accelerator Mass Spectrometry Facility (UCIAMS). The R_Combine function for the three leather samples provides a date range of 3627–3377 Cal BC (95.4% confidence interval) and the calibrated range for the straw is contemporaneous (3627–3377 Cal BC). The shoe was stuffed with loose, unfastened grass (Poaceae) without clear orientation which was more than likely used to maintain the shape of the shoe and/or prepare it for storage. The shoe is 24.5 cm long (European size 37), 7.6 to 10 cm wide, and was made from a single piece of leather that wrapped around the foot. It was worn and shaped to the wearer's right foot, particularly around the heel and hallux where the highest pressure is exerted in normal gait. The Chalcolithic shoe provides solid evidence for the use of footwear among Old World populations at least since the Chalcolithic. Other 4^th^ millennium discoveries of shoes (Italian and Swiss Alps), and sandals (Southern Israel) indicate that more than one type of footwear existed during the 4^th^ millennium BC, and that we should expect to discover more regional variations in the manufacturing and style of shoes where preservation conditions permit.

## Introduction

**Figure 1:**
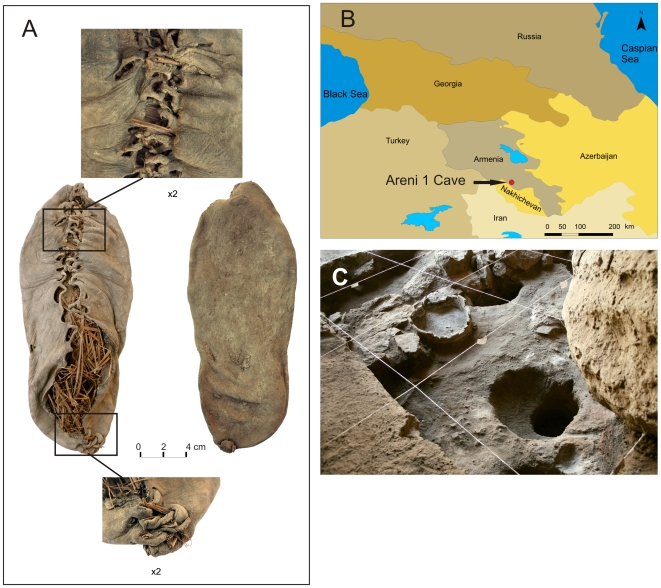
a. Leather shoe from Areni-1, Armenia, b. Map showing location of Areni-1, c. Trench highlighting Pit 3, where the shoe was found.

Knowledge of prehistoric footwear is incomplete and limited to chance finds of well-preserved artifacts. In 2008, a leather shoe (3,653–3,627 cal. BC, Tables 1, 2) was discovered at Areni-1 Cave, Vayots Dzor province, Armenia (39° 43′ 53.4″ N, 45° 12′ 13.4″ E, Figure 1). Desiccated conditions in the cave result in exceptional preservation of organic materials including reeds, ropes, textiles, plant remains and wooden artifacts, providing a rare glimpse into the technology, style, and function of perishable items. To date, this is the oldest shoe discovered in Eurasia. Below we provide the description of the archaeological context, chronology and implications of this discovery to knowledge about the antiquity, function and development of prehistoric footwear.

**Table 1:** Radiocarbon determinations from Areni-1.

	Material	Date	Used (mg)	Yield (mg)	%Yield	%C	δ^13^C	δ^15^N	CN
OxA-20581*	leather	4725±32	53.77	6.75	12.6	39.4	−19.4	5.7	3.5
OxA-20582*	leather	4708±32	62.25	10.39	16.7	42.4	−19.3	5.8	3.3
UCIAMS-65186*	leather	4700±20	nd**	nd	nd	nd	nd	nd	nd
OxA-20583	grass	4810±31	65.38	16.61	25.4	40	−25.1	0.0	0.0

‘Used’ represents the weight (in milligrams) of the amount of starting material used in the pre-treatment chemistry. Yield is the yield of sample in milligrams after pre-treatment. %C is the amount of carbon obtained from the combustion of the treated organic material. For bone we would expect ∼40% by weight to comprise carbon. δ^13^C values are reported in ‰ with reference to VPDB [22], δ^15^N values with respect to AIR. CN represents the atomic ratio of carbon to nitrogen; values between 2.9 and 3.5 would be usual.

*R_Combine dates.

**Table 2:** Calibrated age ranges (BC) according to calibration using INTCAL04 and the OxCal software.

	Calibrated age range (68.2% prob.)		Calibrated age range (95.4% prob.)	
	from	to	from	to
R_Combine (4707,15)	−3618	−3381	−3627	−3377
OxA-20583	−3644	−3535	−3653	−3524
Iceman mean	−3361	−3137	−3366	−3118

**Figure 2:**
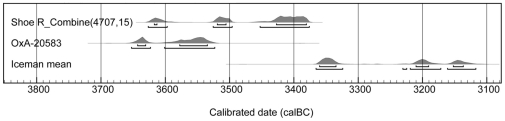
Calibrated age ranges (BC) for the mean radiocarbon age of the three leather determinations (R_Combine) and the determination for the grass sample. These results are compared with the mean value for the determinations obtained from the Iceman.

The ranges represent the total range in age BC. The values for R_Combine are based on the calibration of the mean value for the two leather determinations. See Figure 2 for a diagram of the probability distribution.

Areni-1 is a large karstic cave that contains archaeological cultural strata spanning from the Neolithic to late medieval times. Between 2007 and 2009, excavations were carried out in Trench 1 located deep in the central gallery, Trench 3 in the front gallery near the mouth of the cave, and Trench 4 on the upper part of the slope on the cave's talus. Standard context recording excavation techniques were employed using a Leica Total Station. The upper stratigraphic layers in Trench 3 revealed traces of medieval occupation dated to the 12^th^–14^th^ centuries A.D. with evidence of domestic use of the cave (bread-baking cylindrical ovens, and a distinct assemblage of artifacts such as potsherds of coarse ware and fragments of glass) atop paved and plastered floors of dwellings. A single radiocarbon date of 635±15 BP (KCCAMS 52415, 1293–1392 A.D., calibrated at 95.4%) was obtained from remains of raw cotton (*Gossypium* sp.) from this complex. The underlying stratigraphic layers in both Trench 1 and Trench 3 revealed at least two phases of Chalcolithic occupation in the cave which were radiocarbon dated to the period spanning most of the first half of the 4th millennium BC. The excavations indicate that the Chalcolithic inhabitants used specific parts of the cave for different purposes including habitation, economic, and ritual activities. In Trench 3 Chalcolithic layers revealed dwelling structures and artifacts related to household activities, such as hearths, grindstones, obsidian and chert tools, and animal bones. In contrast, Trench 1 at the rear part of the central gallery was predominantly used for storage and for ritual purposes. The most notable discovery in the rear section was three clay pots each containing one complete subadult skull (burial 1: 8±2 years of age, burial 2: 11±2.5 years of age, burial 3: 15–21 years of age [1]). The antiquity of the skulls was assessed by three radiocarbon dates of teeth to 4330–3990 Cal BC (95.4% confidence interval; Burial 1: OxA 19332, 5323±30 uncal BP; Burial 2: OxA-19331, 5366±31 uncal BP; Burial 3: OxA 18599, 5285±29 uncal BP).

## Results

The shoe was recovered upside down at the base of a shallow, rounded, plastered pit (45 cm deep, 44–48 cm wide) in Trench 3, located beneath an overturned broken Chalcolithic ceramic vessel (Figure 1a, c). Objects found in association with the shoe include a red deer (*Cervus elaphus*) scapula with remains of dried meat adhering to the surface, two complete horns of an adult female wild goat (*Capra aegagrus*) and a fish vertebra placed atop the vessel; a variety of reeds and 40 small ceramic sherds representing 15 different chaff-tempered and grit-tempered vessels, typical of the two Late Chalcolithic occupational phases, were also found within the pit. The shoe was stuffed with loose, unfastened grass (Poaceae) without clear orientation. Ethnographic studies indicate that grasses are often used as wadding to provide warmth and protection [2]. Here, the archaeological context of the shoe and the haphazard orientation of the grasses combined suggest that the grass was used to maintain the shape of the shoe and/or prepare it for storage.

The shoe was worn and shaped to the wearer's right foot, particularly around the heel and hallux where the highest pressure is exerted in normal gait [3]. The shoe is 24.5 cm long (European size 37), 7.6 to 10 cm wide, and was made from a single piece of hide leather that wrapped around the foot. A leather thong was used to stitch the back and top of the shoe through four and 15 sets of eyelets respectively (partially preserved on top with a 2–3 mm diameter; eyelet diameter varies from 0.6 to 1.5 cm). The tension of the frontal thong created interlocking of the left and right eyelets and transverse wrinkles on the vamp. A horizontal thong slit in the upper left side of the instep (only left side preserved) facilitated the fastening of the back part of the shoe to the ankle.

The grain of the leather faces inwards and its mean thickness (measured at six locations) is 2.12 mm (SD = 0.16). The most commonly used animal skins for the production of footwear are those of cow, sheep and goat. The average hide thickness of unprocessed cattle skins is between 4 to 6 mm while those of sheep and goat are between 1 and 2 mm [4]. Unprocessed cattle skin is too thick for shoe uppers and is cut into two layers by the tanner [4]. While the taxonomic fingerprinting of the leather type requires further analysis it appears that the Areni-1 shoe leather was made from a processed cow-hide.

Using forensic charts for the estimation of sex on the basis of foot and shoe dimensions (employing data from modern adult Turkish men and women [5]), it appears that the shoe length is close to the average dimension for females (24.99±1.31 cm) and out of the male range (25.00–32.50 cm). The shoe width is well within the range for adult males (7.00–13.40 cm) and females (5.00–12.20 cm) as well as adolescent males. While there are no similar comparative studies for other ethnic groups, it is known that various populations differ in forefoot shape [6] and that inter-population differences in shoe design exist [7]. An anthropometric study of US Army soldiers born between 1911 and 1970 indicated ethnic differences in secular change in foot length, with a recorded increase among Caucasians and Hispanics, no change among Afro-Americans and a decrease among Asians [8]. Since similar ethnic-specific variation probably also prevailed during the Chalcolithic, it is not possible at this stage to determine with any certainty the sex of the Areni-1 shoe wearer.

Leather from the shoe yielded uncalibrated radiocarbon dates of 4725±32 BP (OxA-20581), 4708±32 BP (OxA-20582), and 4,700±20 BP (UCIAMS-65186). The grass sample dates to 4810±31 BP (OxA-20583). The R_Combine function for the three leather samples provides a date range of 3627–3377 Cal BC (95.4% confidence interval) and the calibrated range for the straw is contemporaneous (3627–3377 Cal BC, Table 2).

## Discussion

Prior to this discovery, the earliest known shoe in Eurasia was worn by Ötzi, the Iceman, who has been dated to 3365–3118 Cal BC (R_Combine, 95.4% confidence interval [9]; Table 2). Only parts of the iceman's left and right footwear were recovered and these were interpreted as including an inner ‘sock’ made of grass, and a separate ‘sole’ and ‘upper’ made of deer and bear leather held together with a leather strap [10]. Reinterpretation of these badly preserved remains suggests that the footwear was a moccasin-type one-piece leather shoe within which the instep attached to an upper ‘sock’ with leather strings in a manner similar to historical footwear of Inuit and Native Americans [3]. Various older sandals, moccasins and slip-on footwear were recovered from Arnold Research Cave, Missouri. There the earliest specimens are sandals made from fiber and/or leather, the oldest of which (specimen 2) date to 7420±50 uncal. years BP (β103270) and 6990±40 radiocarbon years BP (β108745), predating any footwear recovered in the Old World. The earliest slip-on shoe (sample 5) dates to 4680±50 radiocarbon years BP (β103271), rendering it slightly younger than the Areni-1 shoe [11]. In Israel, a pair of worn cow-hide sandals was recovered in association with a Late Chalcolithic male human burial wrapped in shrouds and paraphernalia (at the Cave of the Warrior, Judean Desert [12]. The sandals were not directly dated but associated linen fabrics, straw and reed mats were dated to the first part of the 4^th^ millennia BCE [13] and were thus panecontemporaneous with the Areni-1 find.

It is important to note that both the Iceman's footwear and those from Arnold Research Cave differ from most prehistoric European footwear known to date as they are made of relatively soft leather and lack a vamp. One-piece cow-hide shoes with a vamp have been found across Europe, including Bronze Age Ronbjerg Mose, Denmark [2] and at Early Medieval (200–500 A.D.) Drumacoon Bog, Ireland [2], [14]. An additional shoe found on the Aran Islands of Ireland was made using the same manufacturing technology as the Areni-1 shoe. In Ireland, these shoes are known as “Pampooties,” and are reported to last a very short time, typically no longer than one month [14].

Enormous similarities exist between the manufacturing technique and style of one-piece leather-hide shoes across Europe and the one reported here from Areni-1 Cave, suggesting that shoes of this type were worn for millennia across a large and environmentally diverse geographic region. Given the simplicity of these shoes, it is possible that the design and technology of the shoe was independently invented in various locations across Europe and Southwest Asia. The similarity of the cut and lacing is striking, however, so it also plausible that the technology was invented in one place and spread across the region. Currently the shoe from Areni-1 is the oldest of this type and is also the oldest shoe from Eurasia. While these shoes may have been invented in the Caucasus, given the rarity of such finds it is impossible at this stage to assess when and where the first footwear of this type was first developed. It is likely, however, that the earliest footwear predates the Areni-1 shoe significantly.

Recent biomechanical research on pedal phalangeal robusticity among Upper Pleistocene humans [15], [16] suggests that footwear was already in use during the Middle Palaeolithic and became more common during the middle Upper Palaeolithic (∼27,500 cal. BP). The biomechanical analysis shows gracialisation of the middle 3^rd^, 4^th^, and 5^th^ pedal foot phalanges with a retention of robust lower limbs and halluces. Trinkaus [15], [16] suggests that this is the consequence of a reduction in the habitual loads on the forefoot related to the use of footwear which can be traced back to mid-latitude archaic modern humans (Tainyuan I, China). However, footprints in European Upper Paleolithic parietal art show a wide anatomical variation and in general appear to portray unshod feet [15]. Palaeolithic footwear may, therefore, have been either uncommon or perhaps not depicted by means of artistic media. It is possible that prehistoric footwear was predominantly used in order to protect the feet from rugged terrain and to provide warmth. Simple one-piece leather shoes made of relatively thick hide would have provided some insulation, particularly when padded with grasses, and would have easily molded to the anatomical shape and dimensions of the wearer's feet. Such footwear may not, therefore, have induced the same foot pathologies commonly seen among people nowadays who wear leather shoes with a rigid sole [17], [18]. Studies of anatomical variations in foot shape and pressure distribution among shod and unshod populations indicate that the latter tend to have wider feet and more equally distributed peak pressures of the plantar load carrying surface than in habitually shod subjects [19]. A comparative analysis of the frequency of pathological conditions in foot metatarsal bones of recent (Sotho, Zulu and European) and pre-pastoral South African skeletal samples indicate that the foot of the unshod pre-pastoralist group is healthier as mid-foot and other pathologies are rare [20]. However, some pathologies (e.g., hypertrophy of the medial and dorsomedial eminence, dorsal lipping and eroded crista of the first metatarsal head, osteophytes of the bases of metatarsals three, four and five, irregular cortical lesions of the lesser metatarsal shafts) are common in both unshod and shod populations while pathologies of the first metatarsal (predominantly hallux valgus) are common among shod populations [21]. It appears, therefore, that more research is required on the effects of specific types of footwear on various human populations as it is necessary to take into consideration not only intra- and inter-population variations in anatomy and gait, but also variability in environmental and geographic factors (climate, terrain type, etc.).

We can conclude that the Areni-1 one-piece cow-hide shoe provides solid evidence for the use of footwear among Old World populations since the Chalcolithic; more than likely the use of footwear began during much earlier epochs. Both the Ice Man's shoes and the shoe from Areni-1 are relatively simple and sole-less. These finds, taken together with the sandals from the Cave of the Warrior, indicate that more than one type of footwear existed during the 4^th^ millennium BC, and that we should expect to discover more regional variations in the manufacturing and style of shoes where preservation conditions permit. More research investigating the specific pathological and morphological effects of this specific footwear type on male and female foot bones is needed, as this information will allow the assessment of new evidence for its distribution and antiquity.

## Methods

Leather from the Areni-1 shoe was dated at the Oxford Radiocarbon Accelerator Unit, University of Oxford and at the University of California-Irvine Accelerator Mass Spectrometry Facility. Grass from within the shoe was dated at the ORAU. The leather was chemically pretreated using an acid-base-acid sequence. First, the leather was treated with 0.5 M HCl at room temperature (RT), then with 0.2 M NaOH at RT and finally with 0.5 M HCl. Between each step, the leather was rinsed with distilled water. Finally, the sample was treated using a bleach step with 2.5% (w:vol) NaCLO3 at pH 3 at 70°C for ∼30 minutes. The grass sample (OxA-20583) was treated in a similar manner, but the acid-base-acid used 1 M HCl and 0.2 M NaOH at 80°C in each case.

After pre-treatment, all samples were weighed into pre-cleaned tin capsules and combusted in a CHN elemental analyser, operating in continuous flow mode using a He carrier gas linked with a Europa IRMS. δ^13^C values are reported with reference to VPDB [22]. Graphite was prepared by reduction of CO_2_ over an iron catalyst in an excess H_2_ atmosphere at 560°C prior to AMS radiocarbon measurement [23, 34]. All radiocarbon determinations are calculated with reference to Stuiver and Polach [25]. The results are given in Table 1. Calibration of the results was undertaken using OxCal 4.1b3 [26] and the INTCAL04 calibration curve of Reimer et al. [27] (Table 2). We used the R_Combine command to derive a mean for the three leather determinations. The results are shown in Figure 2.

## References

[1] Buikstra JE, Ubelaker DH (1994). Standards for data collection from human skeletal remains..

[2] Hald M (1972). Primitive shoes: an archaeological-ethnological study based upon shoe finds from the Jutland Peninsula..

[3] Goubitz O, van Driel-Murray C, Groenman-van- Waateringe W (2001). Stepping through time: archaeological footwear from prehistoric times until 1800..

[4] Haines BM, Barlow JR (1975). The anatomy of leather.. Journal of Materials Science.

[5] Ozden H, Balci Y, Demirüstü C, Turgut A, Ertugrul M (2005). Stature and sex estimate using foot and shoe dimensions.. Forensic Sci Int.

[6] Hawes MR, Sovak D, Miyashita M, Kang S-J, Yoshihuku Y (1994). Ethnic differences in forefoot shape and the determination of shoe comfort.. Ergonomics.

[7] Işcan MY (2005). Forensic anthropology of sex and body size.. Forensic Sci Int.

[8] Griener TM, Gordon CC (1990). An assessment of long-term changes in anthropometric dimensions: secular trends of US Army males..

[9] Kutschera W, Werner R (2000). Ötzi, the prehistoric Iceman.. Nucl Instrum Methods Phys Res B.

[10] Goedecker-Ciolek R, Egg M, Goedecker-Ciolek R, Groenman-van Waateringe W, Spindler K (1993). Zur Herstellungstechnik von Kleidung und Aüstrustungsgegenständen.. Die Gletschermumie vom Ende der Steinzeit aus den Ötztaler Alpen.

[11] Kuttruff JT, DeHart SG, O'Brien MJ (1998). 7500 Years of Prehistoric Footwear from Arnold Research Cave, Missouri.. Science.

[12] Schick T (1998). The Cave of the Warrior: A Fourth Millennium Burial in the Judean Desert..

[13] Jull AJT, Donahue DJ, Carmi I, Segal D, Schick T (1998). Radiocarbon dating of finds.. The Cave of the Warrior: A Fourth Millennium Burial in the Judean Desert.

[14] Lucas AT (1956). Footwear in Ireland.. County Louth Archaeological Journal.

[15] Trinkaus E (2005). Anatomical evidence for the antiquity of human footwear use.. J Arch Sci.

[16] Trinkaus E, Shang H (2008). Anatomical evidence for the antiquity of human footwear: Tianyuan and Sunghir.. J Arch Sci.

[17] Ashizawa K, Kumakura C, Kusumoto A, Narasaki S (1997). Relative foot size and shape to general body size in Javanese, Filipinas and Japanese with special reference to habitual footwear types.. Ann Hum Biol.

[18] Kusumoto A, Suzuki T, Kumakura C, Ashizawa K (1996). A comparative study of foot morphology between Filipino and Japanese women, with reference to the significance of a deformity like hallux valgus as a normal variation.. Ann Hum Biol.

[19] D'Août K, Pataky TC, De Clercq D, Aerts P (2009). The effects of habitual footwear use: foot shape and function in native barefoot walkers.. Footwear Science.

[20] Zipfel B, Berger LR (2007). Shod versus unshod: The emergence of forefoot pathology in modern humans?. The Foot.

[21] Sim-Fook L, Hodgson AR (1958). A comparison of foot forms among the non-shoe and shoe-wearing Chinese populations.. Journal of Bone and Joint Surgery.

[22] Coplen TB (1994). Reporting of stable hydrogen, carbon and oxygen isotopic abundances.. Pure and Appl Chem.

[23] Bronk Ramsey C, Hedges REM (1997). Hybrid ion sources: radiocarbon measurements from microgram to milligram.. Nucl Instrum Methods Phys Res B.

[24] Dee M, Bronk Ramsey C (2000). Refinement of Graphite Target Production at ORAU, Proceedings of 8th AMS Conference Vienna.. Nucl Instrum Methods Phys Res B.

[25] Stuiver M, Polach H (1977). Discussion: Reporting of 14C data.. Radiocarbon.

[26] Bronk Ramsey C (2001). Development of the radiocarbon calibration program OxCal.. Radiocarbon.

[27] Reimer PJ, Baillie MGL, Bard E, Bayliss A, Beck JW (2004). IntCal04 terrestrial radiocarbon age calibration, 0–26 cal kyr BP.. Radiocarbon.

